# IgM Deficiency in Autoimmune Blistering Mucocutaneous Diseases Following Various Treatments: Long Term Follow-Up and Relevant Observations

**DOI:** 10.3389/fimmu.2021.727520

**Published:** 2021-09-27

**Authors:** A. Razzaque Ahmed, Merve Aksoy

**Affiliations:** ^1^ Department of Dermatology, The Center for Blistering Diseases, Tufts University School of Medicine, Boston, MA, United States; ^2^ Dermatology Service, Boston Veterans Administration Health Services, Boston, MA, United States

**Keywords:** rituximab, post-rituximab, hypogammaglobulinemia, decreased IgM, low IgM, blistering disorder/diseases, immunosuppressive agents, intravenous immunoglobulin

## Abstract

IgM deficiency has been reported in patients with many autoimmune diseases treated with Rituximab (RTX). It has not been studied, in detail, in autoimmune mucocutaneous blistering diseases (AIMBD). Our objectives were: (i) Examine the dynamics of IgM levels in patients with and without RTX. (ii) Influence of reduced serum IgM levels on clinical and laboratory parameters. (iii) Explore the possible molecular and cellular basis for reduced serum IgM levels. This retrospective study that was conducted in a single-center from 2000 to 2020. Serial IgM levels were studied in 348 patients with five AIMBD (pemphigus vulgaris, pemphigus foliaceus, bullous pemphigoid, mucous membrane pemphigoid, and ocular cicatricial pemphigoid) and found decreased in 55 patients treated with RTX, IVIG, and conventional immunosuppressive therapy (CIST). Hence the incidence of decreased serum IgM is low. The incidence of decreased IgM in patients treated with RTX was 19.6%, in patients treated with IVIG and CIST, it was 52.8% amongst the 55 patients. IgM levels in the post-RTX group were statistically significantly different from the IVIG group (p<0.018) and CIST group (p<0.001). There were no statistically significant differences between the groups in other clinical and laboratory measures. Decreased serum IgM did not affect depletion or repopulation of CD19+ B cells. Patients in the three groups achieved clinical and serological remission, in spite of decreased IgM levels. Decrease in IgM was isolated, since IgG and IgA were normal throughout the study period. Decreased IgM persisted at the same level, while the patients were in clinical remission, for several years. In spite of persistent decreased IgM levels, the patients did not develop infections, tumors, other autoimmune diseases, or warrant hospitalization. Studies on IgM deficiency in knockout mice provided valuable insights. There is no universally accepted mechanism that defines decreased IgM levels in AIMBD. The data is complex, multifactorial, sometimes contradictory, and not well understood. Nonetheless, data in this study provides novel information that enhances our understanding of the biology of IgM in health and disease.

## Introduction

IgM is an important molecule for many reasons. Interestingly, it is the first immunoglobulin produced as a part of the adaptive immune system, which has evolved over 500 million years (1). The varying and similar structures of the molecule are present in jawed fishes, amphibians, reptiles, birds, and mammals (2). In all these species, it helps discriminate self and non-self, protect against pathogens, and provides the memory of previous infections (3). IgM and IgD are regarded as ancient isotypes and play an important role in the emergence of the significant diversity of the immune response (4, 5).

In spite of its evolutionary and current importance in the immune system, it has not been studied in detail in autoimmune diseases.

A molecule of such phylogenetic importance and one which currently seems to be of significance in the immune response of humans deserves attention. Recently, we published a review of Rituximab (RTX) associated serum IgM decrease in multiple autoimmune diseases (6). While mention of it has been in an earlier study on only pemphigus vulgaris (PV) patients, many critical details were not included (7). Other autoimmune blistering diseases were not included (7). Therefore, we decided to study decreased serum IgM levels in AIMBD in a more detailed comprehensive clinically relevant manner.

In this study, we present data on patients with pemphigus vulgaris (PV), pemphigus foliaceus (PF), bullous pemphigoid (BP), mucous membrane pemphigoid (MMP), and ocular cicatricial pemphigoid (OCP), who had decreased levels of serum IgM, during their systemic therapy.

PV affects the skin and mucosal tissues in the oral cavity, nose, pharynx, larynx, esophagus, genitalia, anal canal, and nails (8). In PF, it is limited to the skin. The blisters are very superficial and rupture easily, leaving denuded surfaces. Histologically the blisters are intraepidermal, and on direct immunofluorescence, the autoantibody binds the surface of epidermal cells (8).

BP manifests as tense blisters predominantly involving only the skin, usually in an acral distribution. The blisters are subepidermal on histology, and the autoantibody binds to the basement membrane zone (BMZ) (9).

MMP involves the mucosa predominantly, less frequently the skin. The mucosa of the nose, oral cavity, conjunctiva, pharynx, larynx, esophagus, genitalia, and anal canal. The most important feature of MMP is that, as the lesions heal, they cause scarring. The blisters are submucosal, and the autoantibody binds to the BMZ. When the disease predominantly involves the eye, causing scarring in the conjunctiva, and possible blindness, it is referred to as OCP (9).

Rituximab is a chimeric monoclonal antibody that targets CD20+ B cells. Recently its use has been associated with decreased levels of serum IgM in several autoimmune diseases. RTX has been used in the treatment of autoimmune diseases that are mediated by autoantibodies or T cells (10–15).

In the study, we present the data on patients with PV, PF, BP, MMP, and OCP who were treated with RTX, intravenous immunoglobulin (IVIG), and conventional immunosuppressive therapy (CIST), and in whom the levels of IgM were followed for long periods of time.

The purpose of this study was to analyze the dynamics of IgM deficiency in these patients and to determine its influence on clinical course, clinical outcomes, relapse rate, and CD19+ B cell levels.

## Methods

### Patient Demographics

The Center for Blistering Disease (CBD) is a tertiary care center for AIMBD patients. This study was approved by the New England Institutional Review Board (IRB).

Hence this is a single-center retrospective study of 55 patients with low Ig M levels seen between 2000 and 2020 at the Center for Blistering Diseases in Boston, MA. The identity or personal characteristics of none of the patients were disclosed.

The following inclusion criteria were used: 1) The diagnosis of diseases (PV, PF, BP, MMP, MMP with OCP) was made on the basis of a combination of clinical presentation, histological features, and every patient had immunopathological confirmation of their diagnosis (8, 9). 2) IgM hypogammaglobulinemia is defined as <50 mg/dl on at least two or more consecutive laboratory testing. (3) Minimum follow up for 12 months or more.

Exclusion criteria included (1) Lack of baseline IgM level recording or at least two consecutive blood sampling tests with IgM deficiency, (2) Patients who were previously treated with Rituximab, and (3) Patients who lacked histopathology and immunopathology confirmation of diagnosis.

**Figure 1:**
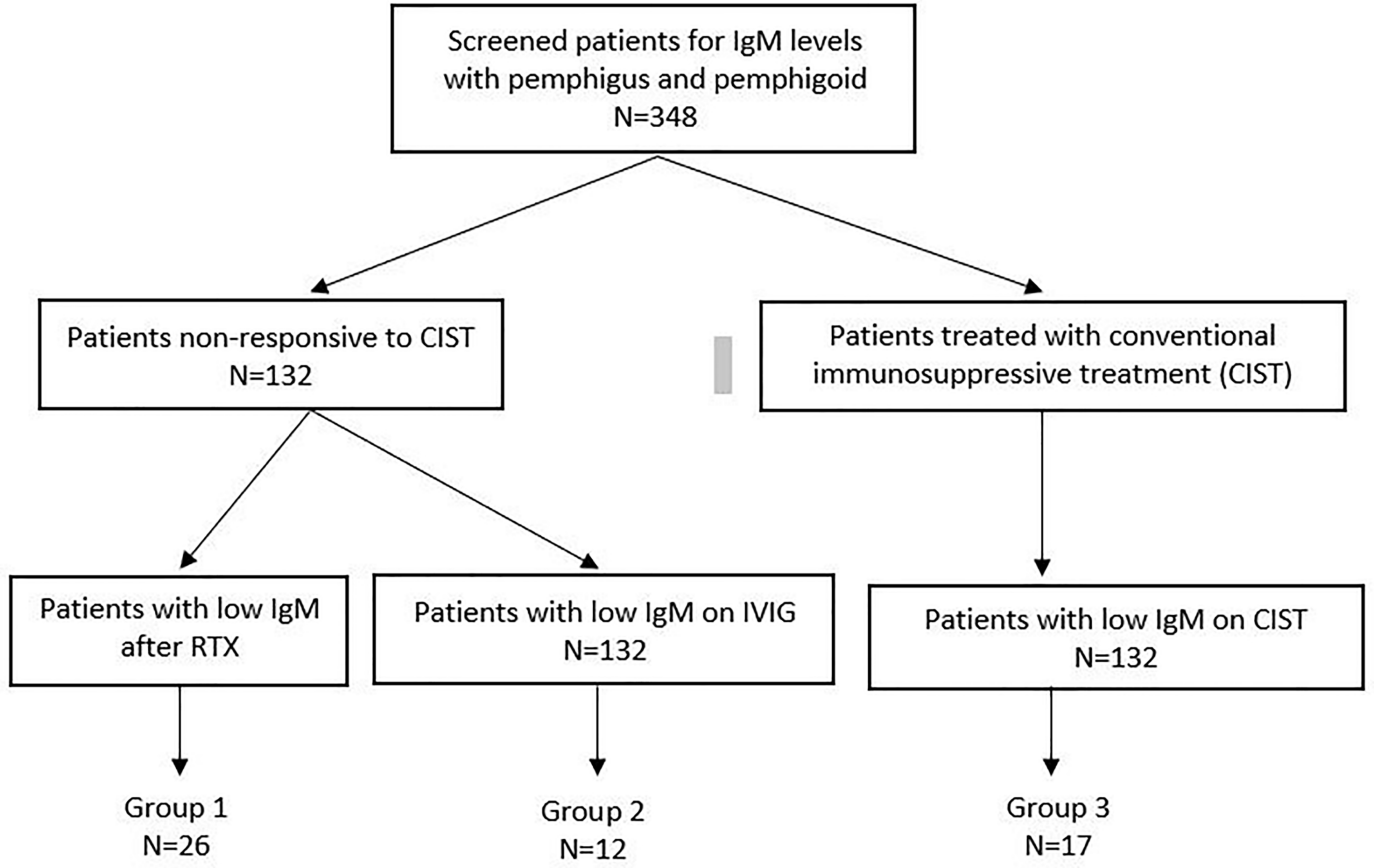
Flow chart diagram demonstrates the number of patients with low Ig M levels in Group 1, 2 and 3 among the patients with available IgM data.

The method by which patient records were screened and patients with low serum IgM levels were identified are present in **Figure 1**.

The patients were divided into three groups. Patients in Group I were treated with RTX. Patients in Group II were treated with IVIG. Some of these patients subsequently got RTX. Patients in Group I and II had moderately severe or severe disease, with multiple sites of involvement. Patients refractory to other therapies were treated with RTX, according to the Ahmed protocol (16). This protocol consisted of 12 infusions of RTX in 14 months and concomitant IVIG. Patients in Group III received only CIST.

The following data were collected on every patient: sex, ethnicity, disease category, age at onset, duration of disease prior to entering the study, and systemic drugs used by patients prior to entering the study (**Table 1**). Clinical outcomes were recorded: as the time to clinical remission and/or relapse, the incidence of SAE (severe adverse event), and infection. The follow-up period was the duration between the initial encounter to last clinical observation.

**Table 1:** Baseline data of the patients Group I, Group II and Group III.

Category	Group I* (N = 26)	Group II** (N = 12)	Group III (N = 17)
Sex (Male %)	13 (50%)	4 (33%)	9 (52.7%)
Ethnicity (%)			
Caucasian	14 (53.8%)	6 (75%)	12 (85.7%)
Hispanic	3 (11.5%)	0 (0%)	0
African American	0 (0%)	1 (12.5%)	0
Jewish	3 (11.5%)	1 (12.5%)	0
Asian	2 (7.7%)	0 (0%)	2 (14.3%)
Type of disease (%)			
Pemphigus vulgaris	12 (46.2%)	3 (25.0%)	6 (35.3%)
Pemphigus foliaceus	2 (7.7%)	1 (8.3%)	1 (5.9%)
Bullous pemphigoid	1 (3.8%)	0 (0%)	4 (23.5%)
Mucous membrane pemphigoid (MMP)	4 (15.4%)	1 (8.3%)	5 (29.4%)
MMP with OCP***	7 (26.9%)	7 (58.3%)	1 (5.9%)
Mean Age at onset (yr)/(Range)	51.6/(32-77)	58.6/(49-80)	59.1/(37-74)
Median duration of disease before the treatment (mo)/(Range)	47.0/(4-156)	22.5/(0-114)	18.0/(2-144)
Drug therapy before entering study (%)			
Corticosteroid	11 (42.3%)	8 (66.6%)	9 (52.9%)
ISA	9 (34.6%)	10 (83.3%)	3 (17.6%)
Azathioprine	1 (3.8%)	2 (16.6%)	3 (17.6%)
Methotrexate	4 (15.4%)	4 (33.2%)	0
Mycophenolate mofetil	3 (11.5%)	3 (25.0%)	0
Tacrolimus	1 (3.8%)	0 (0%)	0
Cyclophosphamide	0 (0%)	1 (8.3%)	0

*Group I was treated with rituximab, according to the Lymphoma protocol, dose 375mg/m^2^, given as a weekly infusion, once a week, for four consecutive weeks.

**Group II was treated with Intravenous Immunoglobulin (IVIg). The dose of IVIg was 2gm/kg/cycle. The frequency of administration was based on clinical response, but dosage remained constant Refs: (i) J Dermatol Sci 2017; 85:77-84 (ii) J. Am. Acad Dermatol 2009; 60: 595-603 (iii) J. Am. Acad Dermatol 2001; 45: 679-690 (iv) J. Am. Acad Dermatol 2001; 45: 825-835 (v) Expert Opinion Investig Drugs, 2004; 13: 1019-1032.

***p value < 0.02, The percentages of the patients in Group II as compared to Group III was different in MMP with OCP disease category. This difference was statistically significant.

### Treatment

CIST consisted of systemic corticosteroids (CS) and nonsteroidal adjuvant therapy such as dapsone or minocycline. Immuno-suppressive agent (ISA) was chosen based on severity, extent, and type of disease. The data is presented in **Table 1**. The number of patients treated with non-steroidal adjuvant therapy included sulfasalazine (6 patients), dapsone (5), and minocycline (4). Immunosuppressive agent (ISA) used in Group I and II were as follows methotrexate (8 patients), azathioprine (6), mycophenolate mofetil (6), tacrolimus (1) and cyclophosphamide (1 patient). This data is present in **Table 1**.

RTX was administered according to the lymphoma protocol at a dose of 375 mg/m^2^, given in four consecutive infusions, once weekly for four weeks. None of the patients were treated with RTX using the rheumatoid arthritis protocol. IVIg was given at a dose of 2gm/kg/cycle. The frequency of the infusion was based on clinical response. This protocol has been used in most of AIMBD’s with significant success and clinical outcomes. It is referenced at the bottom of **Table 1**.

### Clinical and Immunological Evaluation

At the time of initial evaluation, serum IgG, IgM, and IgA were determined in all 348 patients. Laboratory outcomes were measured as follows: the duration and time to IgM deficiency, time to CD19+ B-cell depletion, and repopulation in patients treated with RTX. In these 55 patients, each time IgM was measured serum IgG and IgA were also measured. The rate and time to serologic remission were noted in each patient and compared among the patient groups.

The lymphocyte subset CD19+ B cell levels of depletion and repopulation were defined as <6 cells µ*L* and ≥6 cells µ*L*, respectively. Levels of autoantibodies to Dsg 1 and 3 (For PV and PF), antibodies to BP 180 and 230 BPAg1 (For BP, MMP, MMP with OCP) antibodies were measured prior to initiation of therapy and subsequently periodically assessed to determine serological remission. These assays were done by a commercial laboratory using kits made by MBL Ltd Nagoya Japan.

Serious adverse events and the number of hospitalizations due to disease or as a consequence of its therapy were carefully recorded. Any serious infection that warranted systemic antimicrobial therapy or hospitalization was recorded.

### Autoimmune Diseases

At the time of initial evaluation, in all 348 patients, extensive serological testing was done for a panel of autoantibodies to exclude polyautoimmunity. If all serologies were negative, patients had a CT scan of the neck, chest, abdomen, and pelvis to exclude any possible tumor or latent infection since almost all the therapies used were immunosuppressive.

### Statistical Analysis

Statistical analysis was performed using SPSS version 27. Categorical data defined as proportions of patients were compared with Pearson’s Chi-Squared Test. Normally distributed quantitative data was identified as mean (Range). According to the homogeneity, two groups were compared with independent t-test and Mann-Whitney U test, respectively. One-way ANOVA and Kruskal-Wallis tests were performed for three group comparisons. The levels of P-value and 2-tailed P values less than 0.05 were considered statistically significant.

## Results

### Patient Categories

348 patients with AIBD screened for immunoglobulin levels at CBD. Among these, 132 were treated with Rituximab or IVIG, and 216 were treated with CIST. Low serum IgM levels developed in 17 patients on CIST (Group III), 26 on RTX (Group I), and 12 on IVIG (Group II). The overall incidence of decreased serum IgM is low. In addition the number of patients in the three groups is relatively low and has the potential to negatively influence the interpretation of results.

### Patients’ Characteristics

The demographics of 55 patients with low IgM levels are presented in **Table 1**. Male/Female ratio, ethnicity, age, type of disease, duration of disease prior to CS, and ISA treatment, are similar in these three groups.

### Clinical and Laboratory Data of Group I and II

The comparison of clinical and laboratory data on patients in Group I and II are presented in **Table 2**.

**Table 2:** Laboratory and clinical data of Group I and Group II.

Data	Group I (N = 26)	Group II (N = 12)	P value
Mean time to IgM deficiency (mo)/(Range)	3.0/(1-24)	0	NA
Duration of IgM deficiency (mo)/(Range)	25.6/(3-78)	37.1/(6-117)	NS
Mean time to B-cell depletion (mo)/(Range)	1/(1-1)	1/(1-1)	NS
Time to B-cell repopulation (mo)/(Range)	19.1/(12-36)	16.8/(9-36)	NS
Serologic remission (%)	9 (34.6%)	5 (41.6%)	NS
Time to serological remission (mo)/(Range)	18.0/(3-60)	16.0/(1-60)	NS
Clinical remission (%)	24 (92.3%)	12 (100%)	NS
Mean time to clinical remission (mo)/(Range)	6.0/(3-60)	10.5/(3-24)	NS
Duration of clinical remission (mo)/(Range)	23.8/(3-72)	26.6/(3-105)	NS
SAE infection (%)	1 (3.8%)	1 (8.3%)	NS
Mean follow-up (mo)/(Range)	37.8/(12-99)	54.0/(12-117)	NS

NA, Non-applicable; NS, Non-significant.

### Prevalence of Decrease in Serum IgM

Amongst the 132 patients unresponsive to CIST, the incidence of IgM deficiency was as follows. In Group I treated with RTX, it was 19.6%, in Group II treated with IVIG, it was 9.6%, and in Group III (CIST), it was 7.8%. When the aggregate 55 patients with IgM deficiency were evaluated, 47.2% were on RTX, while 52.8% did not receive RTX.

### Serum IgG and IgA Levels

In all 55 patients, serum IgA levels were normal (Range from 49 to 298), with minor fluctuation. This evaluation was essential for all patients who were treated with IVIG, to exclude IgA deficiency.

In the 348 patients with AIMBD, all were screened for IgG. In 55 patients, IgG levels were determined on multiple occasions and were normal (Range from 52 to 300). Patients who were treated with IVIG had higher than normal levels of IgG because IVIG contains only IgG.

### IgM Levels and B Cell Depletion/Repopulation

**Figure 2:**
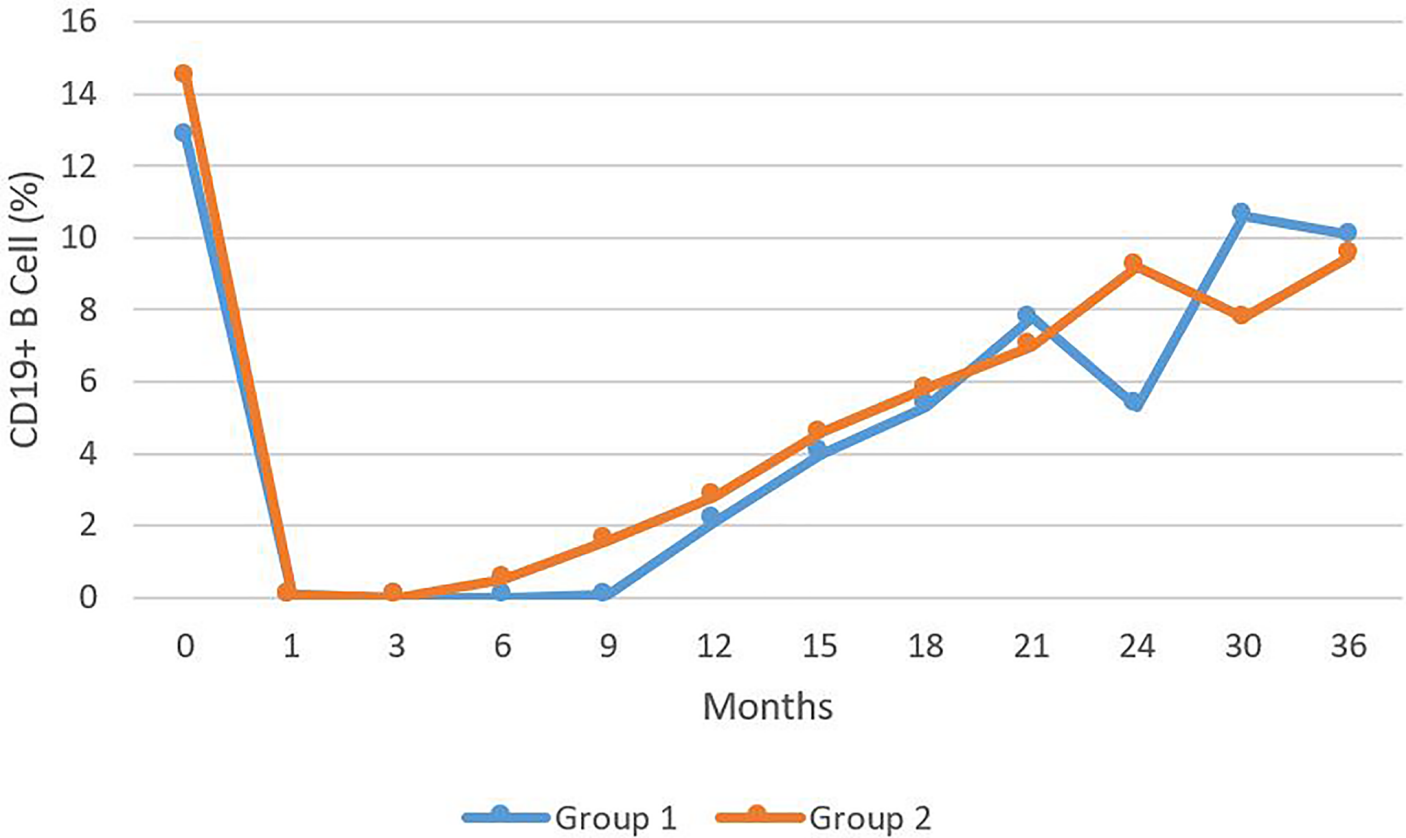
The levels of CD 19+ B cells presents as percentage of total B cell during a 36 month period comparing Group 1 to Group 2. No statistically significant difference was observed.

In all of the 38 patients treated with RTX, B cell depletion began within one month of the initiation of the therapy. B cells remained depleted for a mean of 18 months (Range 12-36) for Group I. IgM deficiency was recorded after three months of therapy (Range 1-24) for Group I. Reduced IgM was already present in Group II, because of earlier treatment with CIST. In Group II, B cells were depleted for a mean of 16 months (Range 9-36) (**Figure 2**). Even after the repopulation of B cells, low IgM levels continued for a mean of 25 months (Range 3-78) for Group I. For Group II, the mean was 37 months (Range 6-117). When statistically compared, the time to develop IgM deficiency and its duration was not statistically significantly different in both groups (p > 0.05) (**Figure 2**).

**Figure 3:**
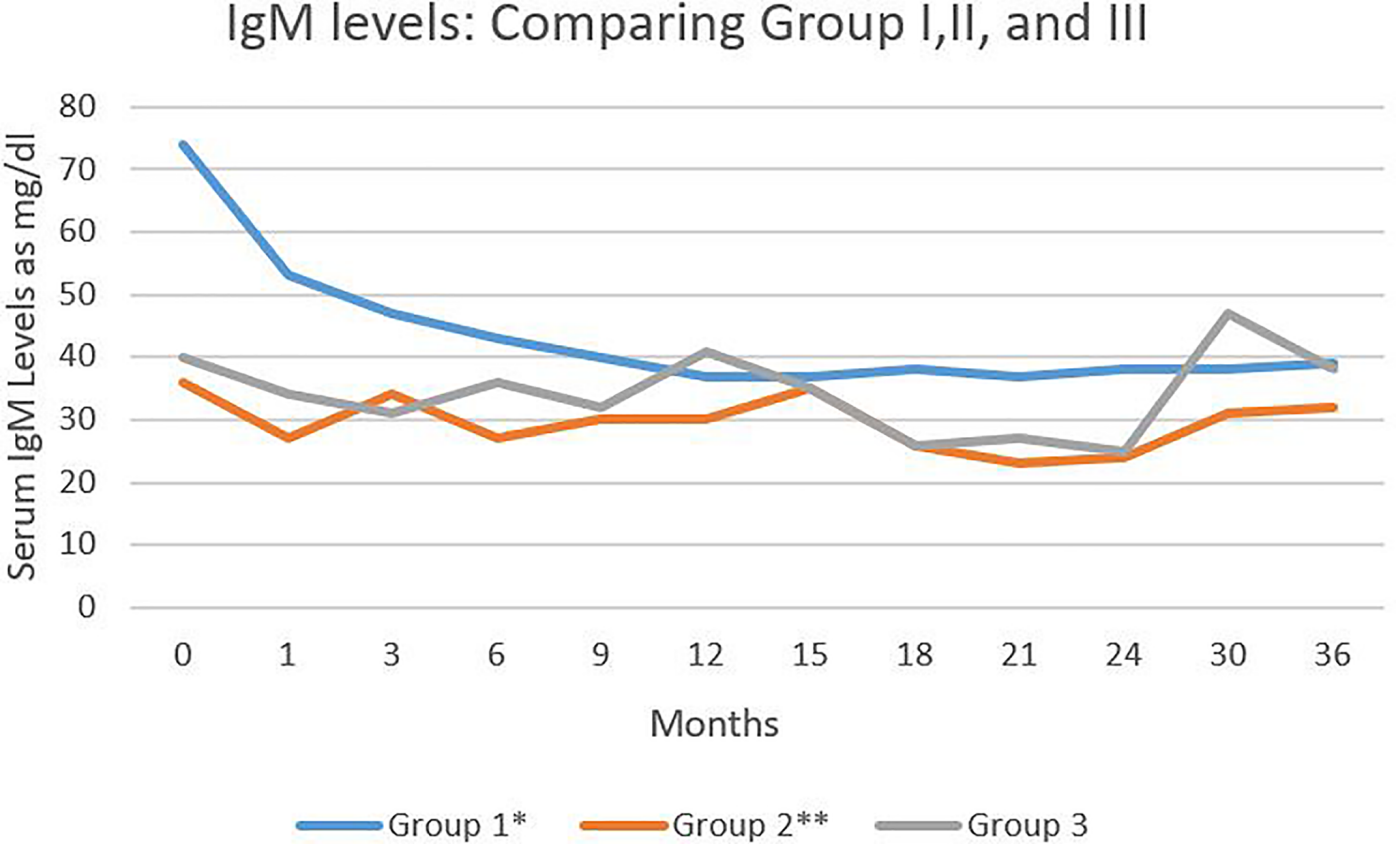
Serum IgM levels in the three study groups during 36 months of follow-up. Serum IgM levels expressed as mg/dl. Comparing group I and III *P < 0.018. Comparing group I and II ** P < 0.01.

The level of IgM in the three groups was statistically significantly different (**Figure 3**). There were statistically significant differences in the levels between Group I and II (p<0.001) and also Group I and III (p<0.018). There was no statistically significant difference between the IVIG and CIST groups.

**Figure 4:**
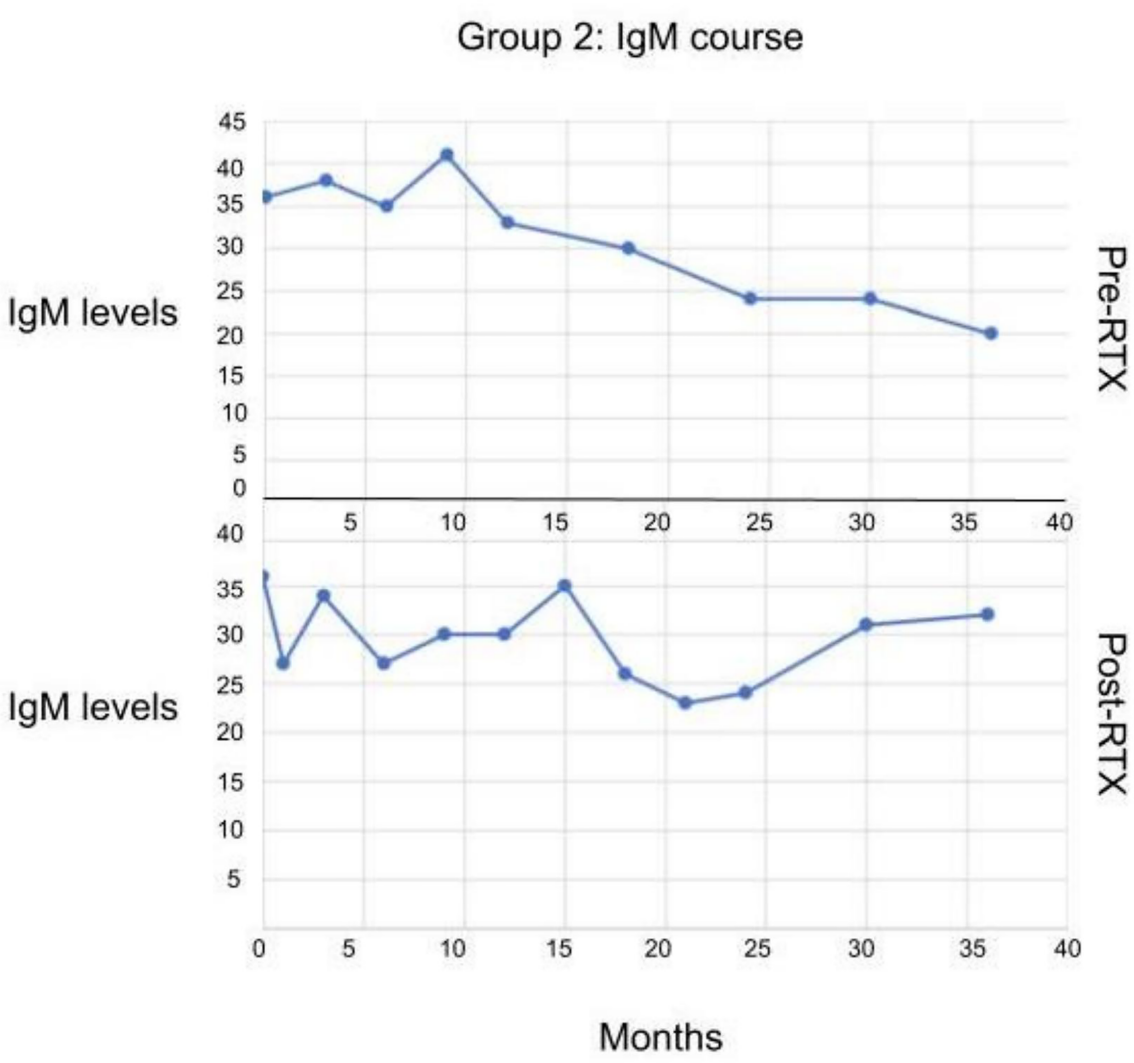
The serum IgM levels in patients in Group II before and after receiving RTX. No statistically significant difference observed.

Some of the patients in Group II who had low IgM initially, when treated with RTX subsequently, the levels of IgM did not decrease further (**Figure 4**).

### Clinical and Laboratory Outcome

The rate of clinical remission in Group I was 92% and 100% in Group II. This high rate of clinical remission in Group II can partially be attributed to the prior use of immunosuppressive agents (ISA). While the ISA did not produce a sustained clinical remission, their pharmacologic actions attributed to the final clinical outcome. In Group I, clinical remission began at a mean of 6 mo (Range 3-60) and was observed for a mean of 23 mo (Range 3-72). In Group II, it began at a mean of 10 mo (Range 3-24) and was observed for a mean of 26 mo (Range 3-105).

The time to serologic remission rate was a mean of 18 mo (Range 3-60) for Group I, 16 mo (Range 1-60) for Group II.

### Side Effects

According to the results, one of 26 (3.8%) patients in Group I and one of 12 (8.3%) patients in Group II had pneumonia during the entire period of observation. It was diagnosed radiologically. It was resolved after oral systemic antibiotic therapy, which was administered at home by visiting nurses and ambulatory care. None of the patients were hospitalized because of their diseases or as a consequence of their treatments (**Table 2**).

### Follow-Up

The follow-up period was a mean of 54 months (Range 12-117) for Group II, and a mean of 37 months (Range 12-99) for Group I (**Table 2**). For Group III, it was a mean of 38 months (Range 12-120).

### Group 3 Clinical and Laboratory Outcome

Since the patients with low IgM levels treated with CIST had a milder disease, they were not included in the comparisons table. The mean time to IgM deficiency was a mean of 13.5 months (Range 6-21), and the mean duration of observation was 22 mo (Range 3-96). The rate of clinical remission in 16 patients was 94.2%, which occurred in a mean of 9 months (Range 1-48). The rate of serologic remission was 52.9% in 9 out of 17 patients. This occurred in a mean of 16 months (Range 3-120). The rate of SAE infection was one of 17 patients (5.9%).

### Autoimmune Diseases

None of the 55 patients in our study group developed a second autoimmune disease. The detailed serologic evaluation did not show the presence of any autoantibodies associated with other autoimmune diseases. All of them were seen by a trained rheumatologist. Repeat serologies were negative. CT scans of the neck, chest, abdomen, and pelvis during remission did not reveal any tumor or infectious process.

## Discussion

In the study, we have provided clinically relevant information on 55 patients treated at the CBD, Boston, MA, with various AIMBD, who developed IgM deficiency.

There are statistically significant differences in the IgM deficiency in patients pre and post RTX therapy and between patients who received RTX compared to those on CIST. There were no significant differences in clinical outcomes, infection rate, and serological remission in all disease categories. Furthermore, IgM deficiency does not influence B cell depletion and repopulation and continues after clinical remission, off all systemic therapy, while CD20+ B cells reach normal pre-treatment levels.

Since 52.8% of 55 patients had low IgM levels without RTX, it can be safely concluded that RTX alone is not responsible for low IgM levels. Low IgM levels are reported in other autoimmune diseases (17). The data does not support the notion that low IgM is a predictor of relapse.

During the follow-up period, some patients treated with RTX had low IgM levels up to twelve years of observation. In other autoimmune disorders such as RA, SLE initially low IgM levels are associated with long-term IgM deficiency (18, 19).

This data demonstrates that the low IgM levels did not influence the duration of CD19+ B cells or the time for repopulation of B cells (CD19+). Interestingly, B cell repopulation to normal levels also did not influence the continued low levels of serum IgM. Only one patient with normal IgM prior to RTX therapy recovered from IgM hypogammaglobulinemia.

It has been reported that the serum levels of IgM decreased with each consecutive infusion of RTX (20). In AIMBD patients, if the serum level of IgM was decreased preRTX, there was not a significant decrease post RTX period. In contrast, AIMBD patients with normal levels of serum IgM did have a significant decrease in serum IgM after RTX.

None of the 55 patients with AIMBD with decreased levels of IgM needed to be hospitalized for infections, during the entire clinical course and follow-up period. This data suggest that there is no increased incidence of infection in IgM deficiency in AIMBD, as reported in other autoimmune disorders (21, 22).

The underlying diseases can affect the prevalence and type of hypogammaglobulinemia (19). The IgM hypogammaglobulinemia prevalence in autoimmune disorders after RTX, ranged from 10% (20) to 58% (21). In AIMBD patients we observed 19%. Low levels of serum IgM are observed in patients with ANCA-associated vasculitis (23).

The influence of drugs on serum IgM levels is multifactorial. The most important variable is the drug, the clinical indication, and when used during the clinical course. Patients with SLE who were treated with RTX and later mycophenolate mofetil developed low levels of IgM (19). Likewise in ANCA-associated vasculitis patients treated with cyclophosphamide and subsequently with RTX developed decreased serum IgM levels (24). Low serum IgM levels are observed in anti-GBM glomerulonephritis treated with azathioprine and prednisone and in primary biliary cirrhosis patients treated with methotrexate (25, 26). In kidney allograft patients, mycophenolate mofetil can cause decreased levels of serum IgM, which return to normal when it was discontinued (27).

There are several limitations in the study. The first is that it is a retrospective study from a single tertiary care center. A control group is lacking. Most importantly, the lack of information on serum IgM levels at the time of diagnosis and before any systemic therapy was initiated, would have provided important insight into the peripheral immune system. Finally, the exact duration of IgM deficiency is not known and the ultimate or final consequence of this long term deficiency of IgM serum is not know (28). During the last follow-up visit, IgM deficiency was still present in many patients. The lack of studies on the bone marrow, spleen, lymph nodes, and peritoneum limit a comprehensive analysis. The authors realize that such studies are unethical and would not be approved by an IRB.

In spite of these limitations, the observations in this study clearly demonstrate that in this cohort of patients with AIMBD, there is a selective decrease of serum IgM. Serum IgA and IgG levels are unaffected. While the frequency differed, all systemic therapy was associated with decreased serum IgM levels. These levels were present when the patient discontinued systemic therapy, were in clinical remission, and it lasted for considerably long durations. They did not after repopulation of CD19+ B cells, and these cells upon reaching normal and pretreatment levels and did not change serum IgM levels. The patients did not suffer from any severe infection or warrant hospitalization. Hospitalization is not very concerning to physicians in many countries for different reasons. In the US lack of hospitalization is critical because the cost of hospitalization is high and affects the overall cost of any therapy. None of the patients developed tumors or other autoimmune diseases. It is very important to note that the serum IgM levels were never undetectable. They significantly decreased but were never zero.

IgM is considered to be a protector, regulator, and scavenger. Membrane-bound Ig M is dimeric, serves as the B cell receptor (BCR), and is important in B cell survival (29–31). Serum IgM is pentameric and occasional hexameric, when the J chain is absent (32). It is shaped with a central protruding region that binds to C1q which is vital for its removal of apoptotic cells. This along with its polyreactivity are the basis for its diversified pathophysiological capabilities.

Reduction of serum levels of IgM raises the important question of which cells produce IgM. Current data suggests that peritoneal B cells (B1a, B1b, and B2) are the main source (33). The bone marrow, which gives rise to the progenitor of conventional (B2) B cells also gives rise to peritoneal (B1) B cells. Consequently, the current understanding is that the compartment and environment in which B cells are present determine their repertoire, phenotype, and ability to secrete IgM (34, 35). Interestingly, B cell subsets can determine the presence of IgM and could increase production to compensate for the decrease (36). Unfortunately, the mechanisms that determine the population expansion of these subsets of B cells are not defined or fully yet understood. Survival of mature B cells is affected by decreased serum IgM. However, survival is impaired in the spleen but enhanced in the peritoneum, suggesting that the importance of niche or microenvironment, in which the B cells reside may be important (37).

Reduction in serum IgM, which promotes survival of B cells in the peritoneum, reduces BCR signaling. Increased peritoneal B cells could provide more autoantibodies (38). This further highlights the complex interaction between IgM and B cell homeostasis in the different compartments.

IgM deficiency predisposes to autoimmunity in humans and mice (39). Apoptotic cells express autoantigens, the persistence of which facilitates the development of autoreactive cells (40). On the contrary, IgM can bind to these molecules and prevent autoimmunity (41). Such opposing data only complicate the explanation of our observations.

One of the other mechanisms for the development of autoimmunity is the altered response to infections which can lead to chronic inflammation by an increased presence of (B1) B cells. This has been demonstrated in mouse models for SLE and diabetes (42, 43). Not surprisingly, removal of the (B1) B cells reduced the severity of the autoimmunity (41).

Relevant to this study and others that use B cell depleting agents, is the observation that peritoneal B cells, seem to be fairly well protected from the B cell depletion, caused by the use of CD20 specific antibodies, such as RTX (44). Low serum IgM observed in patients treated with B cell depleting agents could interfere with apoptotic clearance, and that could explain the lack of response or treatment failure and frequent recurrence or relapse in patients treated with RTX. Clearly, different B cell subsets have different regulatory and pathogenic properties. Serum IgM reduction caused by these therapies may mitigate the benefit, particularly after long term administration, by increasing the risk for infection in patients susceptible to infection, increased risk for atherosclerosis, or even exacerbate autoimmunity.

The addition of IgM to commercially available intravenous immunoglobulin infusion (IVIg) could enhance its efficacy, protection from infection, and autoimmunity. This would help advance our knowledge on IgM and facilitate the development safe and novel therapies.

## Conclusion

In some patients with AIMBD, IgM deficiency is observed. While some readers may consider a cohort of 55 patients with AIMBD a small number, they need to realize two facts. First, these are orphan diseases. In such rare diseases, larger cohorts are not possible. Multicenter or multinational studies are not possible because pharmaceutical companies, who not infrequently fund such studies, would have no interest in reduced serum IgM. Second, these patients were studied in detail and had long term follow-up, which makes these observations meaningful and opens the road for investigators studying more common autoimmune diseases to study IgM deficiency in more detail. Could peritoneal B cells provide protection from infection and prevent autoimmunity?

Factors that predispose a patient to develop IgM deficiency are currently unknown. It does not appear to affect clinical courses or outcomes and persist during remission. The study presents an interesting question. Could autoreactive B cells and/or plasma cells influence IgM synthesis, and what makes this process persist? Another key question that this study raises is whether IgM deficiency is the cause or the effect of autoimmunity and what are the predisposing factors. Investigators of the future may provide valuable insights on this conserved antibody that we know has existed for 500 million years.

## Data Availability Statement

The raw data supporting the conclusions of this article will be made available by the authors, without undue reservation.

## Author Contributions

Both authors (AA and MA) contributed equally to the manuscript. MA collected the database and did the data analysis. AA generated the concept, edited the topic and revised the manuscript. All authors contributed to the article and approved the submitted version.

## Funding

This study was supported in part, by an unrestricted educational grant from the Dysimmune Diseases Foundation.

## Conflict of Interest

The authors declare that the research was conducted in the absence of any commercial or financial relationships that could be construed as a potential conflict of interest.

## Publisher’s Note

All claims expressed in this article are solely those of the authors and do not necessarily represent those of their affiliated organizations, or those of the publisher, the editors and the reviewers. Any product that may be evaluated in this article, or claim that may be made by its manufacturer, is not guaranteed or endorsed by the publisher.

## Abbreviations

AIMBD, Autoimmune mucocutaneous blistering diseases; RTX, Rituximab; PV, Pemphigus Vulgaris; PF, Pemphigus Foliaceus; BP, Bullous Pemphigoid; MMP, Mucous Membranous Pemphigoid; OCP, Ocular cicatricial pemphigoid; BMZ, Basement membrane zone; IVIG, Intravenous immunoglobulin; CIST, Conventional immunosuppressive therapy; ISA, Immunosuppressive agent; SAE, Severe adverse event; NS, Nonsignificant; RA, Rheumatoid arthritis; SLE, Systemic lupus erythematosus.
